# Ectokinases as novel cancer markers and drug targets in cancer therapy

**DOI:** 10.1002/cam4.368

**Published:** 2014-12-14

**Authors:** Garif Yalak, Viola Vogel

**Affiliations:** 1 Harvard Medical School/Harvard School of Dental Medicine, Department of Developmental Biology, Harvard UniversityBoston, Massachusetts, 02115; 2 Laboratory of Applied Mechanobiology, Department of Health Sciences and TechnologyETH Zurich, Switzerland

**Keywords:** Cancer marker, drug design, ectokinases, exokinases, extracellular matrix, extracellular phosphorylation, extracellular protein kinase, personalized medicine

## Abstract

While small-molecule kinase inhibitors became the most prominent anticancer drugs, novel combinatorial strategies need to be developed as the fight against cancer is not yet won. We review emerging literature showing that the release of several ectokinases is significantly upregulated in body fluids from cancer patients and that they leave behind their unique signatures on extracellular matrix (ECM) proteins. Our analysis of proteomic data reveals that fibronectin is heavily phosphorylated in cancer tissues particularly within its growth factor binding sites and on domains that regulate fibrillogenesis. We are thus making the case that cancer is not only a disease of cells but also of the ECM. Targeting extracellular kinases or the extracellular signatures they leave behind might thus create novel opportunities in cancer diagnosis as well as new avenues to interfere with cancer progression and malignancy.

## Introduction

Since the fight against cancer is far from being won, there is a need to think of new strategies to identify alternative targets for cancer diagnosis and combinatorial therapies. Current challenges include the desire to detect cancer much earlier, to prevent or reduce the emergence of acquired drug resistance 1, and to reduce the often lethal side effects. Even more challenging is the fact that different cancer cells from the same tumor can use different pathways to achieve drug resistance 2. The complexity of pathways that can lead to drug resistance prevents to predict which treatment modality might finally allow the host rather the cancer to survive 3,4. Continued chemotherapy will target only a subset of cancer cells, while the resistant cells continue to grow 2. New strategies are therefore needed to target nonresistant and resistant cancer cells. Protein phosphorylation is the key regulatory posttranslational modification exploited for intracellular signaling 5–7, and kinases require sufficiently high ATP levels to transfer a phosphate group. Today, it is believed that one third of human proteins are phosphorylated 8 and small-molecule kinase inhibitors have thus taken the lead as next generation cancer drugs (Table1) 9. While this is a significant progress, these inhibitors often interfere with other complex intracellular signaling networks thus causing sometimes severe side effects, and need to be combined with other approaches.

**Table 1:** Small-molecule kinase inhibitors on the market against kinases

Name	Trade name	Targeted tyrosine kinase	Disease	Producer
Imatinib	Gleevec, Glivec	BCR-Abl	Chronic myelogenous leukemia (CML), gastrointestinal stromal tumors (GISTs), number of other malignancies	Novartis
Gefitinib	Iressa	EGFR	Breast, lung, other cancers	AstraZeneca, Teva
Erlotinib	Tarceva	EGFR	Nonsmall cell lung cancer (NSCLC), pancreatic cancer, several other types of cancer	Genentech, OSI Pharmaceuticals, Roche
Crizotinib	Xalkori	ALK	Nonsmall cell lung cancer (NSCLC)	Pfizer
Dasatinib	Sprycel	BCR/Abl and Src family	Chronic myelogenous leukemia (CML), Philadelphia chromosome-positive acute lymphoblastic leukemia (Ph+ ALL)	Bristol-Myers Squibb
Lapatinib	Tykerb/Tyverb	HER2 and EGFR	Breast cancer, other solid tumors	GlaxoSmithKline
Nilotinib	Tasigna	BCR-ABL, KIT, LCK, EPHA3, EPHA8, DDR1, DDR2, PDGFRB, MAPK11, and ZAK	Chronic myelogenous leukemia	Novartis
Pazopanib	Votrient	c-KIT, FGFR, PDGFR, and VEGFR	Renal cell carcinoma, soft tissue sarcoma	GlaxoSmithKline
Sunitinib	Sutent	PDGF-Rs, VEGFRs, KIT	Renal cell carcinoma (RCC), gastrointestinal stromal tumor	Pfizer
Sorafenib	Nexavar	VEGFR, PDGFR, Raf	Renal cell carcinoma (RCC), unresectable hepatocellular carcinomas (HCC), thyroid cancer	Bayer, Onyx Pharmaceuticals
Vandetanib	Caprelsa	VEGFR, EGFR, RET-tyrosine kinase	Tumors of the thyroid gland	AstraZeneca
Tofacitinib	Xeljanz, Jakvinus	JAK	Rheumatoid arthritis	Pfizer
Ruxolitinib	Jakafi, Jakavi	JAK	Myelofibrosis	Incyte Pharmaceuticals, Novartis

Current FDA-approved kinase inhibitors on the market in cancer treatment.

Cells secrete a cocktail of enzymes, such as cholinesterases, peptidases, transpeptidases, nucleotidases, phosphodiesterases, ectokinases, and ectophosphatases, which lead to posttranslational modifications of extracellular matrix (ECM) proteins, and the composition of this cocktail depends on cell type, external stimulations, and disease 10. Posttranslational modifications of ECM proteins can affect outside-in cell signaling and consequently cell behavior 11. The massive killing of cancer cells typically increases the local extracellular concentrations of the cytoplasmic content, including ATP, thereby causing additional posttranslational modifications of the ECM. The killing of cancer cells will thus leave behind a “diseased” ECM that can send altered instructive signals to the cells that later invade this cancerous ECM left behind. This has not been considered in the treatment of cancer previously.

Beyond using the concentration of extracellular protein kinases in blood to detect cancer in early stages 12–14, ectokinases and ectophosphatases might serve as new drug targets. Shielded by the plasma membrane, drugs with extracellular targets might cause less side effects as they can less directly interfere with intracellular signaling 15–21. Even though cancer is not only a disease of cells but also leads to posttranslational modifications of the ECM, the *intra*cellular focus has overshadowed potential *extra*cellular opportunities that could be exploited to address some of these challenges. Here, we thus review the indications that cancer is not only a disease of cells but also of the ECM, and how this newly emerging knowledge of extracellular posttranslational modifications can potentially be exploited for cancer diagnosis and treatment.

## Extracellular Enzymes and Posttranslational Modifications of ECM Coregulate Cancer Progression

**Figure 1:**
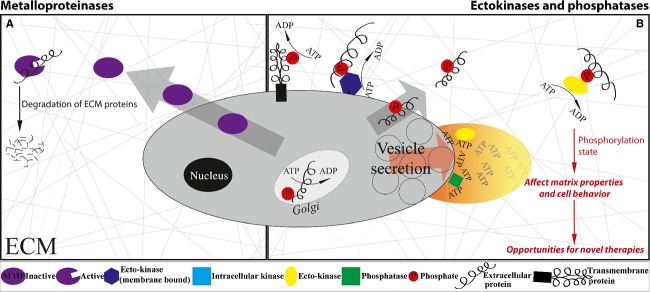
Extracellular enzymes in cancer. (A) To alter the extracellular matrix (ECM) biology of cancer, matrix metalloproteinases (MMPs) served as first extracellular drug targets. The hypothesis was that matrix metalloproteinases (MMP) inhibition can prevent the local degradation of ECM and thus the escape of metastatic stroma cells. (B) Vesicle secretion and cell necrosis transiently releases intracellular content high in kinases and ATP into the surrounding tissue as indicated by the graded plume. For a short time period, the ATP levels are sufficiently high for the kinases to be active in extracellular space leaving behind phosphorylated ECM. Ectokinases and phosphatases thus provide unique opportunities as novel extracellular drug targets.

Extracellular strategies are mostly missing although considerable knowledge emerged that the composition and rigidity of the ECM, and consequently ECM cell signaling plays an important role in cancer progression 22,23. The first wave of targeting ECM enzymes was motivated by the finding that cancer tissues show upregulated matrix metalloproteinase (MMP) levels, and it was thought that MMP-induced cleavage of ECM would promote the escape of cancer cells from the site of tumors 24,25 (Fig.1A). Consequently, MMP inhibitors were designed and went into clinical trials, but with devastating negative outcomes 26,27. The main reason for the failure was the lack of appreciation for the complexity of MMP functions and their respective effects on ECM properties and signaling. Only three MMPs had been described at the time when the clinical trials had started, while 23 different MMPs are known today 28. They were found in different cell types with diverse functions including ECM–cell interactions, cell–cell contact, and regulation of soluble factors among many others 27. Broad-spectrum inhibitions of MMPs thus interfere with their diverse regulatory roles and thereby cause major side effects 29.

It is thus timely to consider alternate extracellular strategies, including extracellular enzymes or other means by which to regulate posttranslational modifications (Fig.1B). While the importance of various posttranslational modifications in the ECM are known to regulate cancer progression 23, the significance of ectokinases and ectophosphatases, and the signatures they leave behind, is only now at the verge of being recognized 30. Why should we even consider extracellular phosphorylation since the ATP levels are typically low in extracellular environment? Extracellular ATP can transiently increase to levels that are sufficiently high to activate ectokinases in those tissues that undergo major necrosis and apoptosis, thereby releasing intracellular content 30. Also ATP secretion pathways are significantly upregulated in cancers 31,32 and increased levels of extracellular ATP could recently been measured at tumor sites 33.

Among the reported *ecto*kinases, the most prominent ones are the casein kinase II (CKII) 34, protein kinase A (PKA) 35, protein kinase C (PKC) 36, and the recently reported Fam20C kinase 37. Several ectophosphatases including alkaline phosphatase 38, tartrate-resistant acid phosphatase (TRAP) 39, and the most recently reported PTEN phosphatase 40 have been reported in the ECM. Interestingly, the concentration of the extracellular alkaline phosphatase is already measured routinely as a disease marker in patient's blood samples to detect liver diseases, bone disorders, or cancer and the TRAP is being discussed as a good candidate 39,41.

Taken together, considerable evidence is emerging that posttranslational modifications of ECM coregulate cancer progression, that ectokinases and ectophosphatases are found in body fluids of cancer patients, and that kinases can be transiently active in extracellular space in regions where necrosis or other factors cause the release of ATP. As with any discovery, new ideas and strategies are thus beginning to emerge how to exploit these emerging insights into early cancer detection and therapy.

## Striking Signatures of Extracellular Kinase Activity Are Found in Cancer Tissues

**Figure 2:**
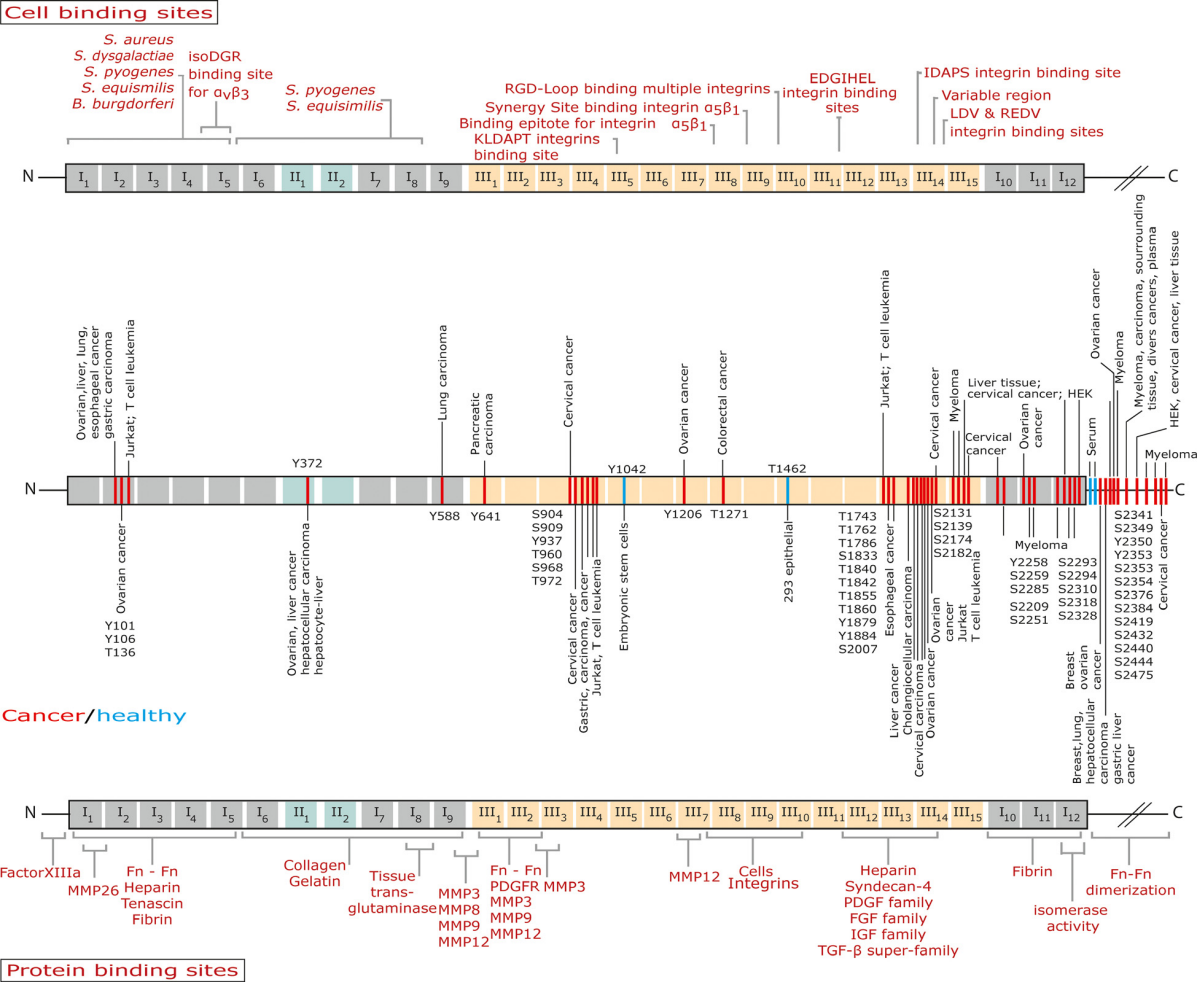
Experimentally verified phosphorylation sites on fibronectin. Schematic representation of plasma fibronectin with modules type I (gray), type II (turquoise), and type III (orange). (A) Locations of various bacterial and cell binding sites on the fibronectin monomer. (B) Experimentally identified phosphorylation sites by mass spectrometry techniques as retrieved from protein data banks Phosida, PhosphoSitePlus, PhosphoNet, HPRD, dbPTM, and UniProt for human Fn (P02751). (C) Locations of protein binding sites on the fibronectin monomer with a special focus on matrix metalloproteinases (MMPs).

Postulating that massive necrosis might temporarily upregulate ectokinase activity in extracellular space, we recently mined published proteomic data and found a significant upregulation of phosphorylated residues in tissue samples from cancer patients 30. This included the phosphorylation of ECM proteins, as well as of cell surface and extracellular domains of transmembrane proteins. Screening more than 60 different extracellular proteins revealed that nearly all can occur in phosphorylated states 30. Most compelling was the finding that the integrin subunits *α*4 and *β*1, two key players in cancer progression and signaling, were found in tissue samples to be phosphorylated in their extracellular domains 30,42–44. Since fibronectin 45–47 which is a key component of the ECM is known to be highly upregulated in cancer 48–53, we further analyzed published proteomic data and found that fibronectin is indeed heavily phosphorylated in clinical cancer tissue samples (Fig.2, Table2). Heavily phosphorylated regions in fibronectin include and are associated with growth factor binding sites (FnIII_4_, FnIII_13-14_) and with domains that regulate fibronectin fibrillogenesis. This is an important finding since growth factor signaling and ECM fibrillogenesis are essential regulators in cancer malignancy and progression 22. In addition to fibronectin, elevated levels of phosphorylated fibrinogen A are found in the plasma from patients with stage III or IV ovarian cancer compared to healthy controls 54.

**Table 2:** Experimentally verified phosphorylation sites on fibronectin in cancer samples

Residue (P02751)	Location/binding sites	Reference/databases	Cancer tissues/cells
Y101, Y106, T136	FnI2, Fn–Fn, Heparin, Tenascin, Fibrin	PhosphositePlus, PhosphoNet	In seven patients samples (Y101): ovarian, liver, lung, esophageal, gastric
			In one patient sample (Y106): ovarian
			In one patient sample (T136): T-cell leukemia
Y372	FnII1, Collagen, Gelatin	PhosphositePlus, PhosphoNet	In three patients samples: ovarian, liver, hepatocellular carcinoma, hepatocyte–liver
Y588	FnI9, Collagen, Gelatin	PhosphositePlus, PhosphoNet	In one patients sample: lung carcinoma
Y641	FnIII1, Fn–Fn	PhosphositePlus, PhosphoNet	In one patients sample: pancreatic carcinoma
S904	Linker FnIII3–FnIII4	Phosida, PhosphositePlus, PhosphoNet, HPRD,dbPTM 79	HeLaS3 (cervical cancer)
S909	FnIII4, DNA binding	HPRD, dbPTM 80	Hela cells
Y937, T960, S968, T972	FnIII4, DNA binding	PhosphositePlus, PhosphoNet	In one patients sample (Y037): gastric
			In one patients sample (T960): T-cell leukemia
			In one patients sample (S968): T-cell leukemia
			In one patients sample (T972): T-cell leukemia
Y1042	FnIII5	PhosphositePlus, PhosphoNet, dbPTM 81	Embryonic stem cells
Y1206	FnIII7	PhosphositePlus, PhosphoNet	In two patients samples: ovarian
T1271	FnIII8, Cell binding region	PhosphositePlus, PhosphoNet	In one patients sample: colorectal
T1462	FnIII10, Cell binding region	PhosphositePlus 82	293 (epithelial)
T1743, T1762, T1786, S1833, T1840, T1842, T1855, T1860, Y1879, Y1884	FnIII13, Heparin, Syndecan-4	PhosphositePlus, PhosphoNet	In one patients sample (T1743): T-cell leukemia
			In one patients sample (T1762): esophageal
			In one patients sample (T1786): esophageal
			In one patients sample (S1833): liver, cholangiocellular carcinoma
			In two patients samples (1840): cervical
			In one patients sample (T1842): cervical
			In two patients samples (T1855): cervical
			In one patients sample (T1860): cervical
			In one patients sample (Y1879): ovarian
			In two patients samples (Y1884): ovarian, T-cell leukemia
S2007	Variable region IIICS, LDV, REDV integrin binding sites	Phosida 83	Hela cells
S2131, S2139	FnIII15, Cryptic cysteine	84	U266 (immortal B lymphocytes derived from multiple myeloma)
S2174	FnIII15, Cryptic cysteine	Phosida, 83,85,86	Hela cells, HEK, human liver tissue
S2182, S2209	FnIII15, Cryptic cysteine	Phosida 83	Hela cells
S2251	FnI10, Fibrin binding	84	U266 (immortal B lymphocytes derived from multiple myeloma)
Y2258	FnI11	PhosphositePlus, PhosphoNet	In one patient sample: ovarian
S2259, S2285, S2293	FnI11, Fibrin binding, Protein-disulfide isomerase binding	84	U266 (immortal B lymphocytes derived from multiple myeloma)
S2294	FnI12, Fibrin binding, Protein-disulfide isomerase binding	Phosida, 83,85,86	Hela cells, HEK, human liver tissue
S2318	FnI12, Fibrin binding, Protein-disulfide isomerase binding	dbPTM 84	U266 (immortal B lymphocytes derived from multiple myeloma)
S2328	FnI12, Fibrin binding, Protein-disulfide isomerase binding	Phosida, 83,85,86	Hela cells, HEK, Human liver tissue
S2341, S2349	C-terminus, Disulfide bonds for Fn assembly	dbPTM, 84	Serum
Y2350	C-terminus, Disulfide bonds for Fn assembly	PhosphositePlus, PhosphoNet	In two patients samples: breast, ovarian
Y2353	C-terminus, Disulfide bonds for Fn assembly	PhosphoNet, PhosphositePlus 87	In 12 patient samples, breast, lung, gastric, liver, hepatocellular carcinoma
S2353	C-terminus, Disulfide bonds for Fn assembly	Phosida, 83,85,86	Hela cells, HEK, human liver tissue
S2354	C-terminus	PhosphositePlus, PhosphoNet	In one patient sample: ovarian cancer
S2376	C-terminus, Disulfide bonds for Fn assembly	84	U266 (immortal B lymphocytes derived from multiple myeloma)
S2384	C-terminus, Disulfide bonds for Fn assembly	Phosida, PhosphoSitePlus, PhosphoNet, dbPTM, UniProt, 83,84,86,88–90	In 14 patients samples: breast, skin, liver, hepatocellular carcinoma, and surrounding tissue, blood plasma U266 (immortal B lymphocytes derived from multiple myeloma), Hela cells
S2419	C-terminus, Disulfide bonds for Fn assembly	83,85,86	Hela cells, HEK, human liver tissue
S2432, S2440	C-terminus, Disulfide bonds for Fn assembly	HPRD 84	U266 (immortal B lymphocytes derived from multiple myeloma)
S2444	C-terminus, Disulfide bonds for Fn assembly	Phosida 83	Hela cells
S2475	C-terminus, Disulfide bonds for Fn assembly	HPRD 83,85,86	Hela cells, U266 (immortal B lymphocytes derived from multiple myeloma)

Phosphorylated sites by mass spectrometry retrieved from protein databases. Due to lack of track changes and updates of the databases, the reported sites here may differ from the database entries at later points. Table as of Nov. 2014.

Taken together, available data suggest that the upregulated phosphorylation of fibronectin and of some other extracellular proteins is a distinct signature of cancerous ECM. The phosphorylation of the ECM caused by the transient release of ATP by dying cells might thus be physiologically far more important in regulating cancer cell differentiation and tumor progression than previously thought. Indeed, the phosphorylation ratio of peptides increase with tumor size as has been previously shown 13. Any discovery of new signatures how cancer or cancer tissues are different from the norm might offer valuable entrance points for novel diagnostic or therapeutic strategies. Furthermore, extracellular proteins that are highly phosphorylated in some but not in other cancer types might be suitable as novel markers for the early detection of cancers, or perhaps serve as signature of its malignancy.

## New Strategies for Cancer Diagnostics: Quantification of the Concentrations and Activities of Extracellular Protein Kinases

**Figure 3:**
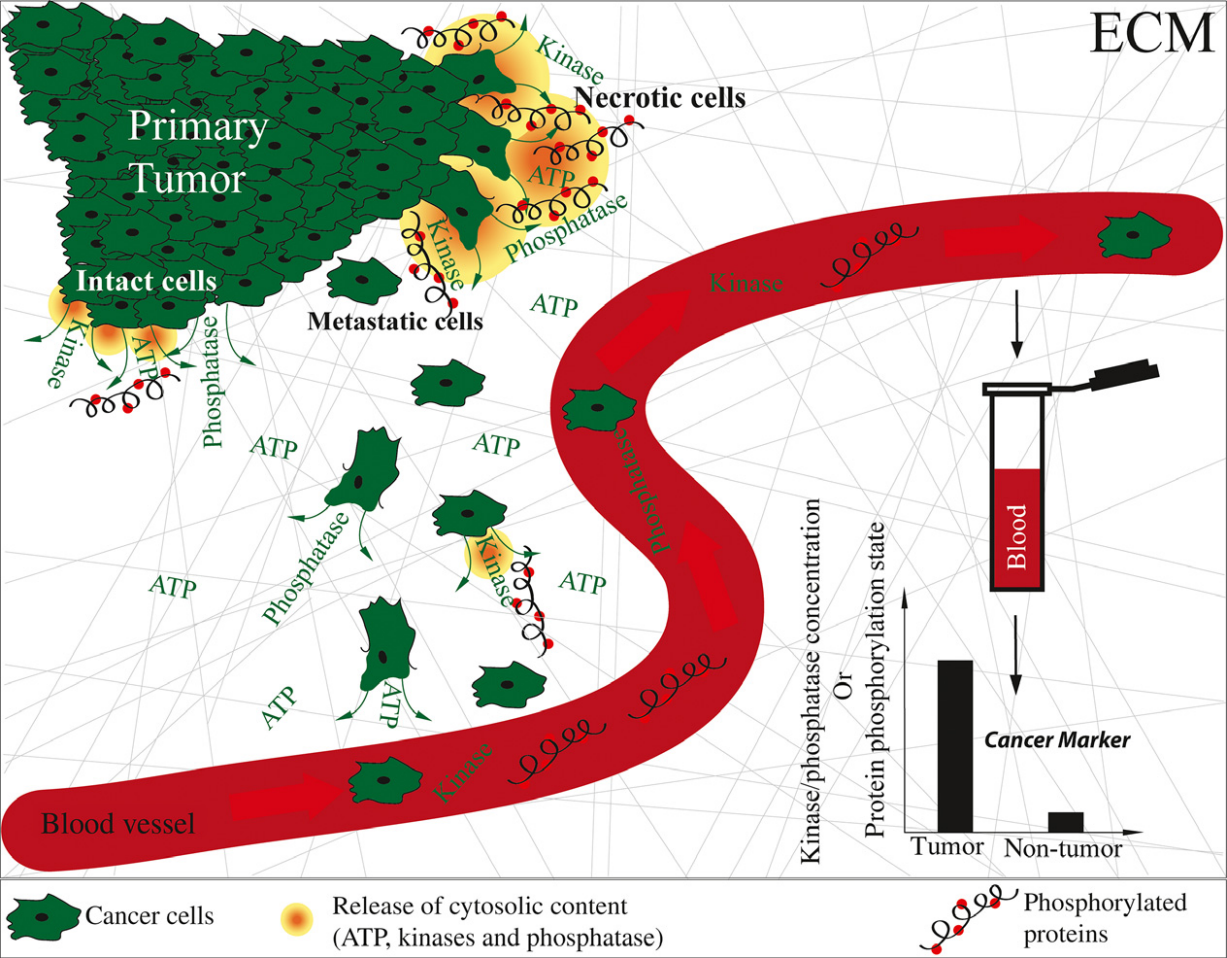
From tumor sites to the blood stream: ectokinases and phosphatases as cancer markers. Enhanced ectokinase and ectophosphatase secretion is seen in intact tumor cells as well as from dying necrotic cells. Blood samples from cancer patients have significantly enhanced ectokinase and ectophosphatase concentrations and activities 64 and might thus serve as novel biomarkers. Several studies report higher selectivity and specificity of markers for early stage cancer detection.

One major challenge is to detect cancer in earlier stages in order to treat patients more successfully. According to recent cancer statistics, the 5-year survival rate dramatically drops if cancer is detected at a late stage 55. Most of the current serum tumor markers are based on the antigen determination method, including CEA, AFP, hCG, PSA, and CA125, but lack tumor specificity and often cannot be used in early cancer screening and diagnosis 56–63. To overcome these shortcomings, novel, cheap, and fast diagnostic tools need to be developed. Measurements of ectokinase and ectophosphatase concentrations and activities in serum might thereby provide new opportunities (Fig.3). Such measurements could be embedded in routinely performed blood tests to screen for cancer long before patients show symptoms. Recent studies with more than 600 patients (374 healthy controls, 229 cancer patients) showed a significant upregulation of ecto-PKA concentrations in serum of cancer patients in contrast to healthy controls 64. While more than 70% of the control patients had undetectable or low ecto-PKA concentrations in serum, more than 85% of the cancer patients had high levels of PKA concentrations, with average activity fivefold higher compared to the healthy controls. In another independent study, sera of 500 patients (295 various cancers, 155 normal controls, 55 without cancer) were analyzed by autoantibody against ecto-PKA. The presented anti-ecto-PKA measurement showed a 90% sensitivity and 80% specificity compared to the conventional methods with 83% sensitivity and 80% specificity 65. Only recently, the quantification of ecto-PKA has been patented as a cancer marker for prostate and breast cancer 66. As suggested by the research group, this approach has the potential to replace the commonly used PSA screening test for prostate cancer and the mammograms screening test for breast cancer, which cost nearly $6 billion annually in the United States alone, with limited reliability of the outcome 67–69. Alternatively or in combination, the ecto-PKC and ecto-CKII are other kinases well suited for phenotyping as they are reported in the ECM and show upregulated levels in secretory vesicles of prostate cancer samples 36,70. Such a path holds considerable promise particularly since a 10-fold increased abundance of ecto-PKC in serum of cancer patients with renal, colon, rectal, adrenal, and lung cancer compared to normal serum has recently been reported 71.

## New Strategies for Cancer Treatment: Drug Targeting of Extracellular Protein Kinases and Phosphatases

In the last two decades, intracellular protein kinases have emerged as the most important drug targets in pharmaceutical industry leading to some 20 approved drugs on the market and hundreds more in clinical trials 72. To reduce side effects, a combinatorial approach is needed, one targeting and killing cancer cells while one also tries to prevent or revert the diseased state of ECM. One can further speculate that these drugs might have less side effects as they will not directly interfere with intracellular signaling events 73, but are expected to regulate primarily outside-in cell signaling. Since several important intracellular protein kinases and phosphatase including PKA, PKC, CKII, FAM20C, alkaline phosphatase, and PTEN phosphatase have been found as ectokinases and ectophosphatases, especially in cancer malignancy and progression 30, their potential as novel drug targets has been highlighted 74,75, but not yet systematically exploited. The overexpression of ecto-PKA in secretory vesicles in prostate cancer further points to a putative regulatory role of ectokinases in cancer 70. The expression of the ecto-PKA kinase, as probed in serum of melanoma patients, correlated with the appearance and size of the tumor and tumor removal reduced the levels of ecto-PKA 14. Ecto-PKC is another kinase that has been reported to be present and active in sera of cancer patients with renal, colon, rectal, adrenal, and lung cancer 36,71. Both ecto-PKC and ecto-CKII have been reported to be expressed in secretory vesicles in prostate cancer and they might thus serve as novel targets 70. The role for FAM20C kinase 37, which is present and active in the ECM, is already discussed in the regulation of bone metastasis 76.

Besides protein kinases, protein phosphatases could also serve as potential drug targets. Most recently, monoclonal antibodies were designed to target the extracellular alkaline phosphatase that is expressed on the surface of gastrointestinal cancer cells 77. In addition, the PTEN phosphatase, a tumor suppressor that is known to induce tumor cell death in vitro and in vivo, has been reported to be secreted and subsequently enter other cells where it modifies their signaling and survival 40. Finally, mutations in PTEN and their down regulation are reported to be involved in invasion and metastasis of colorectal carcinomas, indicating PTEN as a novel drug target and a marker for colorectal carcinoma 78. Another advantage is that ectokinases and ectophosphatases could be targeted in cases where other drugs are not efficient anymore due to resistance of the tumor. Consequently, selected extracellular protein kinases and phosphatases might be good candidates for the development of novel drug targets.

## Future Perspectives

As extracellular protein phosphorylation is moving into the spotlight of attention, our goal here is to stimulate a thinking process how to best utilize this information for the fight of cancer. An increased understanding of the role of ectokinases and ectophosphatases in the regulation of outside-in signaling pathways in cancer malignancy and progression might result not only in exciting new science but also in the design of new combinatorial drugs that can display their functions in extracellular space 15–21, perhaps complementing conventional therapies, by modulating outside-in cell signaling through the posttranslational modification of extracellular proteins. Starting to apply the knowledge gained in the last 60 years about *intra*cellular protein kinases to the extracellular space offers new opportunities. Ultimately, we need to learn not only how to effectively kill cancer cells but also how to repair diseased cancerous ECM that is left behind and has the potency to send altered instructive signals to newly invading cells.
